# Supplementary figures and images for: ROCK Inhibitor (Y-27632) Abolishes the Negative Impacts of miR-155 in the Endometrium-Derived Extracellular Vesicles and Supports Embryo Attachment
Source: Cells. 2022 Oct 10;11(19):3178. doi: 10.3390/cells11193178 (PMC9564368; doi:10.3390/cells11193178)

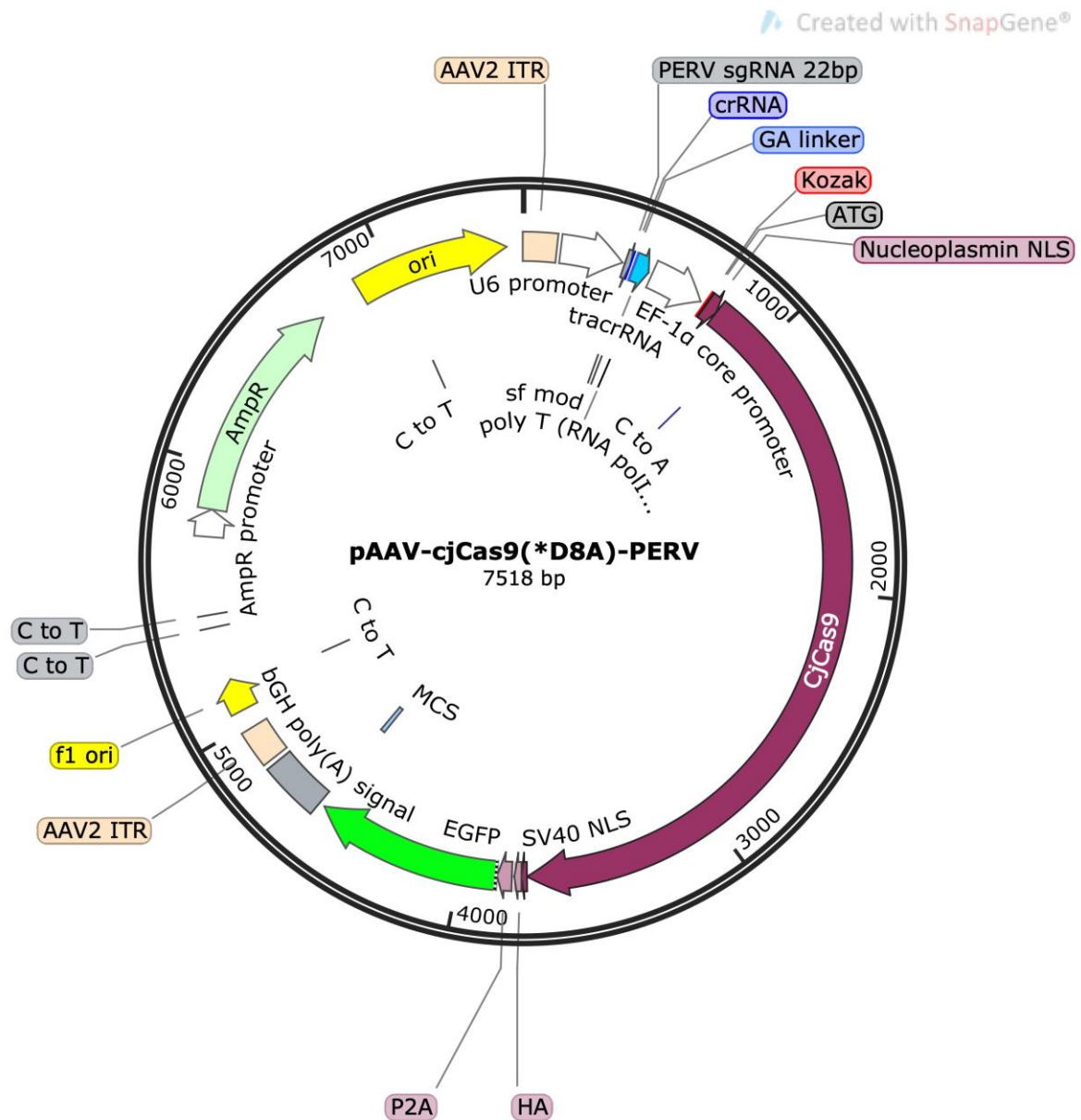

**Figure S1.** The vector used for depletion of PERV through Crispr/Cas9.

Supplement: Supplementary file 1 [file cells-11-03178-s001.zip › Figure S1. vector.pdf]
